# Short Lysine-Containing Tripeptide as Analgesic Substance: The Possible Mechanism of Ligand–Receptor Binding to the Slow Sodium Channel

**DOI:** 10.3390/life14101337

**Published:** 2024-10-21

**Authors:** Vera B. Plakhova, Arina D. Kalinina, Nadezhda A. Boichenko, Dmitriy M. Samosvat, Georgy G. Zegrya, Irina P. Butkevich, Viktor A. Mikhailenko, Valentina A. Penniyaynen, Svetlana A. Podzorova, Roza I. Yagudina, Boris V. Krylov, Ilya V. Rogachevskii

**Affiliations:** 1 Pavlov Institute of Physiology, Russian Academy of Sciences, 199034 Saint Petersburg, Russia; verapl@mail.ru (V.B.P.);; 2 Ioffe Institute, Russian Academy of Sciences, 194021 Saint Petersburg, Russia; 3 Department of Organization of Medical Provision and Pharmacoeconomics, Sechenov First Moscow State Medical University (Sechenov University), 119991 Moscow, Russia

**Keywords:** lysine-containing peptides, Na_V_1.8 channel, patch-clamp method, organotypic tissue culture method, formalin test, conformational analysis, docking, nociception, analgesics

## Abstract

A possible molecular mechanism of the ligand–receptor binding of Ac-Lys-Lys-Lys-NH_2_ (Ac-KKK-NH_2_) to the Na_V_1.8 channel that is responsible for nociceptive signal coding in the peripheral nervous system is investigated by a number of experimental and theoretical techniques. Upon Ac-KKK-NH_2_ application at 100 nM, a significant decrease in the effective charge carried by the Na_V_1.8 channel activation gating system Z_eff_ is demonstrated in the patch-clamp experiments. A strong Ac-KKK-NH_2_ analgesic effect at both the spinal and supraspinal levels is detected in vivo in the formalin test. The distances between the positively charged amino groups in the Ac-KKK-NH_2_ molecule upon binding to the Na_V_1.8 channel are 11–12 Å, as revealed by the conformational analysis. The blind docking with the Na_V_1.8 channel has made it possible to locate the Ac-KKK-NH_2_ binding site on the extracellular side of the voltage-sensing domain VSD_I_. The Ac-KKK-NH_2_ amino groups are shown to form ionic bonds with Asp151 and Glu157 and a hydrogen bond with Thr161, which affects the coordinated movement of the voltage sensor up and down, thus modulating the Z_eff_ value. According to the results presented, Ac-KKK-NH_2_ is a promising candidate for the role of an analgesic medicinal substance that can be applied for pain relief in humans.

## 1. Introduction

The universal language of the brain is the language of nerve impulses. In the 1920s, it was discovered that an increase in the stimulus intensity resulted in an increase in the discharge frequency of an afferent fiber innervating feline mechanoreceptors. The input–output function of the primary afferent fiber describes the relationship between the stimulus intensity and the number and frequency of evoked action potentials [1]. The “sixth” sensory modality, pain, up to now attracts special attention of physiologists and clinicians. It is difficult to overestimate the significance of attempts to control the mechanisms of pain sensation in order to achieve practical results regarding pain relief in humans. The steps in this direction have been performed by researchers who laid the foundations of nociception as one of the most important branches of sensory physiology [2]. The International Association for the Study of Pain characterizes chronic pain as an unpleasant physical sensation and emotional experience that is associated with actual or possible damage and lasts beyond the normal healing period of 3 months [3]. The treatment of chronic pain remains an urgent problem because there are no safe analgesics in the arsenal of practical medicine, the long-term use of which would not lead to negative side effects. Thus, the results of the Global Burden of Disease Study 2013 showed that in 188 countries of the world that participated in this survey, pain syndromes occupy a leading place among all diseases. The majority of respondents were demonstrated to have been experiencing pain for 10 years, but in some cases, the respondents have been in pain for 30 years. The most common types of pain are back pain (46.5% of all respondents), headaches (22.6%), joint pain (21.4%), cranial neuralgia (5.9%), neuropathic pain (3.1%), and psychogenic pain (0.5%) [4,5].

We propose a new approach to develop safe and effective analgesics. On the one hand, according to the current data, the Na_V_1.8 channels are the molecular markers of nociceptive neurons [6]. On the other hand, we have earlier suggested [7,8] that the high-frequency component of the impulse activity of a nociceptive neuron, which carries information about pain effects to the central nervous system, depends not only on the Na_V_1.8 channel’s density, but also on the value of the effective charge (Z_eff_) carried by the Na_V_1.8 channel activation gating system. A decrease in the Z_eff_ value results in the “shutdown” of the nociceptive component of the neuron firing response. It has been shown that short arginine-containing peptides are able to modulate the Na_V_1.8 channel activation gating system, which might lead to pain relief [9]. The formation of the ligand–receptor complex between the attacking peptide and the channel requires that the peptide molecule contains three positively charged guanidinium groups of arginyl residues, which are located at a distance of 9–12 Å from each other [9]. These functional groups are suggested to be involved in intermolecular ionic bonds with the negatively charged aspartate and/or glutamate carboxylate anions of the Na_V_1.8 channel structure upon the peptide binding. Therefore, a reasonable assumption would be that not only short arginine-containing peptides but also short lysine-containing peptides can efficiently bind to the Na_V_1.8 channel due to the positively charged amino groups in the lysine side chains. In this work, we are testing the hypothesis that it is the short peptide consisting of three lysine amino acid residues that can serve as an analgesic medicinal substance due to its specific interaction with the Na_V_1.8 channel activation gating device.

The current work is aimed at studying the molecular mechanism by which the short lysine-containing tripeptide Ac-Lys-Lys-Lys-NH_2_ (Ac-KKK-NH_2_), an analog of the earlier investigated short arginine-containing tripeptide Ac-Arg-Arg-Arg-NH_2_ [10], modulates the Na_V_1.8 channel functional activity. The following research methodology has been applied: (1) the experimental electrophysiological patch-clamp method is used to study the effect of Ac-KKK-NH_2_ on the Na_V_1.8 channel voltage sensitivity at the molecular level and further evaluate the Z_eff_ change after exposure of the nociceptive neuron to the substance under study; (2) the experimental organotypic tissue culture method is applied to investigate the Ac-KKK-NH_2_ effect on the growth of dorsal root ganglia (DRG) neurites at the tissue level; (3) the behavioral experiments in vivo (formalin test) make it possible to verify the analgesic effect of the tripeptide; (4) theoretical conformational analysis of the Ac-KKK-NH_2_ molecule allows determining the distances between the amino groups of the lysine side chains; and (5) finally, docking of Ac-KKK-NH_2_ with the Na_V_1.8 channel molecule is applied to locate the possible binding sites of the tripeptide. The methodological approach combining the indicated experimental and theoretical methods allows us to elucidate the subtle mechanisms of ligand–receptor binding of short lysine- and arginine-containing peptides with the Na_V_1.8 channel molecule, which results in an analgesic effect at the organismal level.

## 2. Materials and Methods

### 2.1. Chemicals and Reagents

All chemicals, excluding Ac-KKK-NH_2_, were purchased from Sigma (Sigma-Aldrich, St. Louis, MA, USA). Ac-KKK-NH_2_ was synthesized in the Verta Research and Production Company (St. Petersburg, Russia) by the method of classic peptide synthesis using reagents from Sigma (Sigma-Aldrich, St. Louis, MA, USA) and Iris Biotech GmbH (Marktredwitz, Germany) and further characterized with high-performance liquid chromatography (purity of more than 95%) and mass spectrometry.

### 2.2. Patch-Clamp Method

#### 2.2.1. Dissociated Sensory Neuron Culture

The patch-clamp experiments were conducted on dissociated sensory neurons obtained by short-term culturing. Sensory neurons were extracted from 6 spinal ganglia located in the L_5_–S_1_ region of the spinal cords of 3–6-day-old *Wistar* rats and placed in Hanks’ solution. Depending on the animal age, the neurons were subjected to enzymatic treatment at 37 °C for 5–8 min. The enzymatic treatment solution was composed of 1 mL of Hanks’ solution, 1 mL of Eagle’s medium, 2 mg/mL of collagenase type 1A, 1 mg/mL of pronase E, and 1 mM of HEPES Na, pH = 7.4. To purify the cells from the enzymatic solution, the spinal ganglia were centrifugated for 1 min at 900 rpm. The supernatant fluid was replaced with Eagle’s medium supplemented with fetal bovine serum (10%), glucose (0.6%), gentamicin (40 U/mL), and L-glutamine (2 mM). The neurons were further mechanically dissociated by pipetting. The cell suspension was diluted with the culturing medium to achieve the required density of neurons, and visually detectable components of the ganglion connective tissue were manually removed from the solution using a pipette. Isolated neurons were cultured on the surface of 40 mm collagen-coated Petri dishes at 37 °C for 1–2 h. Dissociated sensory neurons were used to experimentally register the Na_V_1.8 sodium currents [9].

#### 2.2.2. Experimental Solutions

Two types of solutions were used for the experiments: the extracellular solution, which filled the experimental bath containing the cells, and the intracellular solution, which filled the microelectrodes. The extracellular solution consisted of 70 mM choline chloride, 65 mM NaCl, 10 mM HEPES Na, 2 mM CaCl_2_, 2 mM MgCl_2_, and 100 nM tetrodotoxin (TTX), pH = 7.4. The intracellular solution contained 100 mM CsF, 40 mM CsCl, 10 mM NaCl, 10 mM HEPES Na, and 2 mM MgCl_2_, pH = 7.2. NaOH and HCl solutions were used to adjust the pH values. As both solutions did not contain potassium cations, all potassium currents were thus eliminated, while calcium currents were blocked with the fluoride anions. TTX was used to block TTX-sensitive sodium channels, which made it possible to exclusively record the responses of the Na_V_1.8 channels.

#### 2.2.3. Registration of Na_V_1.8 Currents

The patch-clamp method was implemented in the “whole-cell recording” configuration using a hardware–software setup, which included a patch-clamp L/M-EPC 7 amplifier, analog-to-digital and digital-to-analog converters (DACs), and a PC with the custom software package for the automated running of experiments developed by us.

The microelectrodes were made from soft borosilicate glass [11] with the help of a P-97 micropipette puller. The resistance of a single microelectrode was 1–2 MΩ, providing a series resistance (R_S_) value not exceeding 3 MΩ during the experiment. The glass electrode was filled with the intracellular solution and brought to the neuron surface using a micromanipulator to create the gigaseal contact between the micropipette and the neuron membrane. To implement the “whole-cell” configuration, a section of the cell membrane under the pipette was broken through by creating negative pressure, while the appearance of capacitive components of the ion current with a large amplitude was observed.

The resting potential inside the microelectrode was −60 mV relative to the potential of the extracellular solution, equal to zero. To avoid noise and vibrations, as well as to freely change the extracellular solution after the gigaseal contact has been established, the studied neuron was lifted above the bottom of the experimental chamber using a micromanipulator. The registration of sodium currents began after a few minutes, since it takes time for the intracellular solution to replace the medium inside the neuron. The minimal experiment duration was 15 min, since this amount of time is empirically shown to be necessary and sufficient for the statistically significant effect to occur, the formation of a ligand–receptor complex between the studied substance and the Na_V_1.8 channel. The maximal experiment duration for a single cell was 60 min.

The operational scheme of the software package used to automatically run the experiments is as follows. Firstly, a sequence of rectangular voltage steps of different magnitude and duration to be applied to the neuron was set, depending on the type of experiment. The sodium currents arising in response to this sequence were registered, visually controlled, and express processed to construct the current–voltage characteristics and the voltage dependence of the chord conductance of the sodium currents G_Na_(E). The next stage was the construction of the logarithmic voltage sensitivity L(E) using the Almers method [12] to determine the Z_eff_ value (in elementary charge units) of the Na_V_1.8 channel activation gating system.

The series resistance R_S_ was evaluated automatically during the experiment to monitor that its value would not exceed 3 MΩ. This parameter determines both dynamic and stationary errors of the patch-clamp method, and its value should be maintained under 3 MΩ. Otherwise, the stationary and kinetic parameters of the currents are obtained with large errors, as demonstrated by theoretical analysis of limitations of the patch-clamp method applicability [13], which results in an incorrect Z_eff_ evaluation. Another error, also dependent on the R_S_ value, is associated with the accuracy of measuring the transmembrane potential difference (E). The actual E value determines the position of the stationary characteristics of the Na_V_1.8 channels relative to the voltage axis. The stationary error (ΔE) can be easily estimated from the expression ΔE = I_Na_^max^ × R_S_, where I_Na_^max^ is the maximum amplitude of the sodium current. The ΔE determines the right shift of all voltage-dependent functions along the E axis. If I_Na_^max^ ≤ 1 nA, the stationary error can be ignored, as it does not exceed 1 mV. In addition to R_S_, the following parameters were also evaluated during the experiment: membrane capacitance (C_m_), capacitive currents (I_C_), and leakage currents (I_L_). Both I_C_ and I_L_ were subtracted automatically.

In our experiments, “peak” current–voltage characteristics were recorded, which made it possible to plot the voltage dependence of chord conductance G_Na_(E) and determine Z_eff_, the effective charge of the Na_V_1.8 channel activation gating system. The Z_eff_ evaluation is the main goal of the patch-clamp investigation, since a decrease in this parameter that controls the Na_V_1.8 channel voltage sensitivity leads to the modulation of the Na_V_1.8 channel functional activity, which should result in pain relief [8]. The loss of cell viability in the course of an experiment was controlled by such indicators as leakage current conductance, membrane capacitance, and series resistance. A “hyperimpulse” protocol was used to evaluate C_m_ and R_S_. This protocol triggers the generation of single stimuli in a range of negative potentials that do not activate sodium currents, thus allowing evaluating I_C_ and I_L_. To calculate the R_S_ value, the leakage current time constant τ obtained for the first 3–5 points of its attenuation phase is used as follows: R_S_ = τ/C_m_, which must be less than 3 MΩ for a neuron to remain in a functional state. If the R_S_ value of 3 MΩ was exceeded, the experiment was terminated.

The program of voltage impulses used to construct the peak current–voltage characteristics consisted of the following stages: the first impulse was equal to the resting potential of −60 mV, then a step of −110 mV was generated. Next, a series of consecutive test impulses with a duration of 50 ms in 5 mV increments were recorded in the voltage range from −60 to 45 mV. In response to each step of the potential, the peak sodium current values were recorded and further used to construct the peak current–voltage characteristics. The reversal potential (E_r_) was determined as the intersection point of the right branch of the current–voltage characteristics with the voltage axis E.

#### 2.2.4. The Almers Method

The effective charge Z_eff_ of the Na_V_1.8 channel activation gating system was calculated using the modified Almers method. The family of slow Na_V_1.8 currents differs from the fast Na_V_1.1 currents, in such a way that the inactivation function of the former is shifted to the right along the voltage axis E. Hence, the amplitude of slow Na_V_1.8 currents depends only on the behavior of the activation gating system, while the inactivation variable is close to unity. Therefore, the Almers method makes it possible to reliably evaluate Z_eff_ using the experimentally registered peak amplitude values of the Na_V_1.8 currents. This method can be applied for the Na_V_1.8 channels, but it is not fit for the fast Nav1.1 channels, since the gating current amplitude in this case depends on the inactivation variable, which differs from unity at the moment when the current reaches its amplitude value.

The ratio of the number of open Na_V_1.8 channels (N_o_) to the number of closed channels (N_c_) is calculated as

$$\frac{N_{0}}{N_{c}}=\frac{G_{N_{a}}\left(E\right)}{G_{Na}^{max}-G_{Na}\left(E\right)},$$

where G_Na_^max^ and G_Na_(E) are the maximal value and the voltage dependence of the chord conductance, respectively. The G_Na_(E) function was obtained experimentally using the patch-clamp method as follows:

$$G_{Na}\left(E\right)=\frac{I_{ampl}(E)}{E-E_{Na}},$$

where I_ampl_(E) is the amplitude value of the sodium current, E_Na_ is the reversion potential for sodium ions. G_Na_(E) is a monotonic function that approaches its maximum value G_Na_^max^ at positive E. According to the Almers theory

$$\underset{E\to -\infty }{lim}\left(\frac{N_{0}}{N_{c}}\right)=\underset{E\to -\infty }{lim}\left(\frac{G_{N_{a}}\left(E\right)}{G_{Na}^{max}-G_{Na}\left(E\right)}\right)\to C*\exp\left(\frac{Z_{eff}\times e_{0}\times E}{k\times T}\right),$$

where C is a constant, e_0_ is the electron charge, k is the Boltzmann constant, T is the absolute temperature.

In the case when the membrane potential tends to minus infinity (E→−∞), Z_eff_ can be evaluated from the tangent of the slope of the asymptote, which passes through the first experimental points determined by the most negative values of E. The limit of this function tends to a one-exponential dependence described by the Boltzmann distribution, which allows making the transition from a macroscopic parameter, the chord conductivity of a homogenous ensemble of Na_V_1.8 channels, to a microscopic parameter, the effective charge carried by the activation gating device of a single Na_V_1.8 channel. This transition is justified by the passage to the limit upon the construction of the Almers function, the logarithmic voltage sensitivity L(E), determined further as follows:

$$L\left(E\right)=\ln\left(\frac{G_{N_{a}}\left(E\right)}{G_{Na}^{max}-G_{Na}\left(E\right)}\right).$$

The asymptote is constructed from the first points of the L(E) function obtained at the most negative values of E, which makes it possible to calculate the Z_eff_ value, which is linearly proportional to the tangent of the slope of the asymptote [12].

### 2.3. Organotypic Tissue Culture

**Figure 1:**
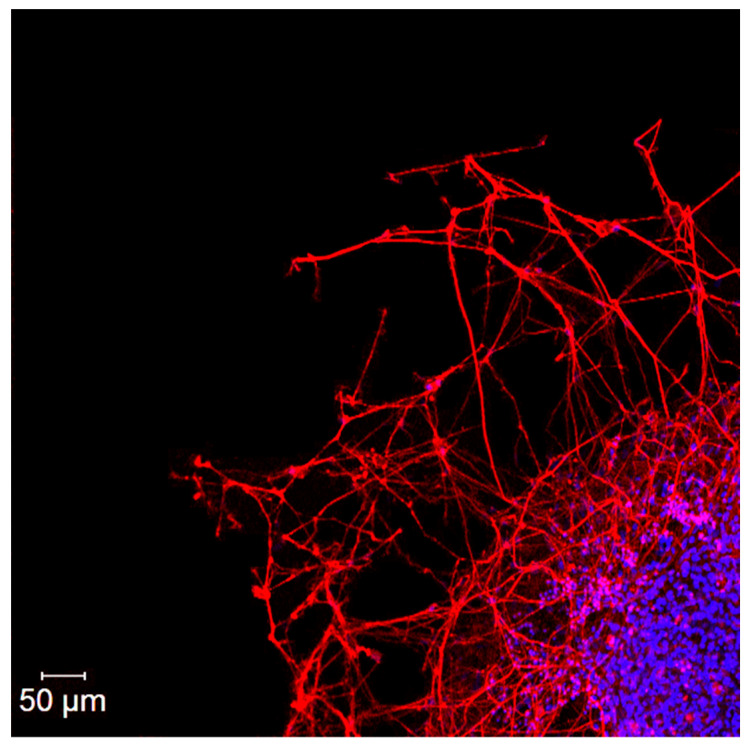
A fragment of control DRG explant growth zone on the third day of culturing. DRG explant is immunostained with anti-neurofilament 200 antibody (red), while DAPI (blue) is used to counterstain the neuron nucleus. Scale bar 50 μm.

The Ac-KKK-NH_2_ effect on the neurite growth of DRG explants from 10–12-day-old *White Leghorn* chicken embryos was studied according to the protocol described in detail earlier [9,14]. In short, the DRG explants were placed in sterile collagen-coated Petri dishes, which were then filled with 3 mL of culture medium and incubated for three days in a CO_2_ incubator (Sanyo, Osaka, Japan) at 37 °C and 5% CO_2_. The culture medium (pH = 7.2) contained low-glucose Dulbecco’s modified Eagle’s medium (1 g/L glucose) supplemented with fetal bovine serum (10%), gentamicin (100 U/mL), and Ac-KKK-NH_2_ at the studied concentration. The control explants were cultured in the same medium but without Ac-KKK-NH_2_ (Figure 1). After incubation, the explants were visualized using an Axio Observer Z1 microscope (Carl Zeiss, Oberkochen, Germany) and analyzed with ImageJ (National Institutes of Health, Bethesda, MD, USA) and ZEN_2012 (Carl Zeiss, Germany) software to evaluate the DRG neurite growth using the area index (AI) calculated as the ratio of the peripheral growth zone area to the central zone area. The average AI value in the control explants was taken as 100% [9,14]. Experiments were conducted using the equipment of the Confocal Microscopy Collective Use Center at the Pavlov Institute of Physiology of Russian Academy of Sciences.

### 2.4. Formalin Test

This test is designed to register the behavioral nociceptive response of rodents to continuous pain induced by tissue damage with formalin, an inflammatory agent [15,16]. In response to the formalin injection, two phases of pain develop. The first, acute short pain, lasts for 5–10 min from the beginning of the injection, and it is associated with the activation of nociceptors and fibers. The second, tonic prolonged pain, begins 15 min after the injection and lasts for 30–40 min, which is associated with the inflammatory reaction. The substances mediating the first phase are substance P and bradykinin; the second, histamine, serotonin, prostaglandins, and bradykinin [16]. It is worth noting that opioid analgesics block both phases of the nociceptive response, nonsteroidal anti-inflammatory drugs block only the second phase, and local anesthetics block only the first phase [17]. Formalin is recommended to be injected into the hind paw, since the hind paws are rarely involved in the natural grooming of rats, which provides a more reliable registration of pain reactions [16,18]. The experiments were conducted on 7 experimental and 7 control adult male *Wistar* rats (average animal weight 220 g). Administration of Ac-KKK-NH_2_ to experimental rats at a dose of 1.0 mg/kg (in Hanks’ solution) and of saline solution to control rats in a volume of 1 mL was performed intraperitoneally 5 min before the formalin injection (2.5%, 50 µL, subcutaneously) into the pad of the left hind paw. Registration of behavioral patterns of the formalin-induced nociceptive response, flexing + shaking (spinal level) and licking (supraspinal level), was carried out for 60 min after formalin had been administered.

### 2.5. Conformational Analysis

The TINKER 8.0 software package [19] with the MMFF94 force field [20] was used to perform conformational analysis of Ac-KKK-NH_2_. The solvation effects were implicitly taken into account in the GB/SA approach [21] at the dielectric constant ε = 10, which simulates the dielectric properties of the environment at the time of ligand–receptor binding of the peptide to the Na_V_1.8 channel molecule, and ε = 80, which simulates physiologically adequate conditions. The low-mode conformational search (LMOD) algorithm was implemented with approximately 100,000 single searches for each structure [22]. The amino groups of the lysine side chains have always been positively charged. The measure of the distance between two amino groups is the distance between their nitrogen atoms. Statistical data processing was performed using our custom C++ script for the entire ensemble of ~100,000 conformations and several subensembles that contained all conformations with energies not exceeding a certain cutoff value relative to the global minimum. In total, 6 subsystems based on cutoff values of 3, 4, 4.5, 5, 6, and 7 kcal/mol were created. The data processing methodology was described in more detail earlier [10].

### 2.6. Peptide Docking with the Na_V_1.8 Channel Molecule

The human Na_V_1.8 channel molecular structure obtained by cryogenic electronic microscopy (PDB code 7WE4) [23] was used. The bound ligand A-803467 was removed, hydrogen atoms were added, and the entire structure was minimized in UFF forcefield [24] using OpenBabel 2.4. All Ac-KKK-NH_2_ conformations from the 7 kcal/mol cutoff subensemble at ε = 10 that had the RMSD value less than 1.5 were selected for docking with the Na_V_1.8 channel.

$$RMSD=\sum_{all\;amino\;groups\;pairwise\;combinations\;i,j}\sqrt{{\left(r_{ij}\right)}^{2}-{\left(\mu_{ij}\right)}^{2}}$$

In the above expression, the summation is carried out over all three pairwise combinations (i =1, j = 2; i =1, j = 3; i =2, j = 3) of the Ac-KKK-NH_2_ lysine amino groups, where r_ij_ is the interatomic distance between the corresponding amino group nitrogen atoms in a given tripeptide conformation, μ_ij_ is the average interatomic distance between the same nitrogen atoms obtained earlier in the conformational analysis. There is no information available regarding the Ac-KKK-NH_2_ possible binding site, so each of the selected conformations was blindly docked 4 times into the entire Na_V_1.8 channel molecule using AutoDock Vina [25] and further analyzed with AutoDockTools [26]. The exhaustiveness was set at 16.

### 2.7. Statistical Analysis

The data were analyzed with STATISTICA 10.0 (StatSoft, Inc., Tulsa, OK, USA) using Student’s *t*-test and expressed as the mean value ± SEM. Statistical significance was set at *p* < 0.05.

## 3. Results

### 3.1. Patch-Clamp Method

**Figure 2:**
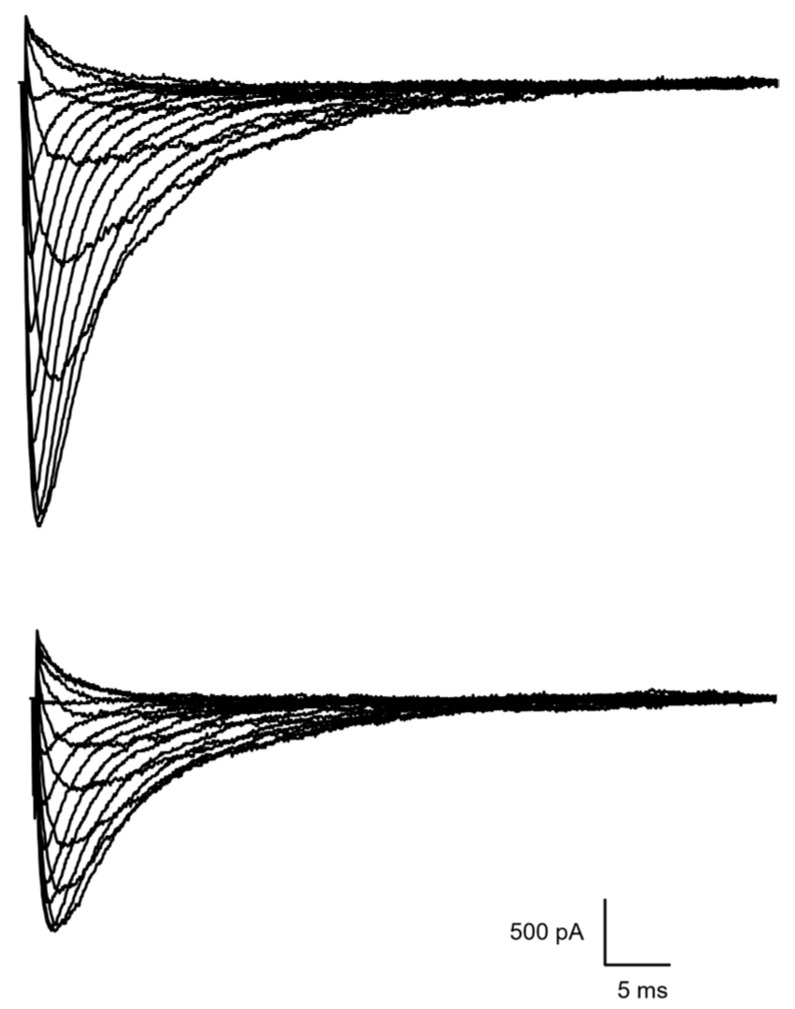
Families of slow sodium Na_V_1.8 currents in the control experiment (**above**) and after extracellular application of Ac-KKK-NH_2_ at 100 nM (**below**). The leakage and capacitive currents were subtracted automatically.

The patch-clamp method was used to study the effects of Ac-KKK-NH_2_ on the Na_V_1.8 channel voltage sensitivity. At the first step, the families of slow sodium Na_V_1.8 currents were recorded under control conditions and after extracellular application of Ac-KKK-NH_2_ at 100 nM. The amplitudes of the Na_V_1.8 currents have decreased as compared to the control, which is due to the rundown effect inherent to the patch-clamp method (Figure 2). The effect is attributed to a gradual decrease in the Na_V_1.8 channels’ density in the neuron membrane caused by functional deterioration of the perfused cell under study. This process, however, does not affect the value, the effective charge of the Na_V_1.8 channel activation gating system. The change in the Z_eff_ value in comparison with the control data reflects the process of ligand–receptor binding of the attacking tripeptide to the Na_V_1.8 channel, and Z_eff_ is the fundamental quantitative parameter that describes the effect of Ac-KKK-NH_2_ on the Na_V_1.8 channel functional activity.

**Figure 3:**
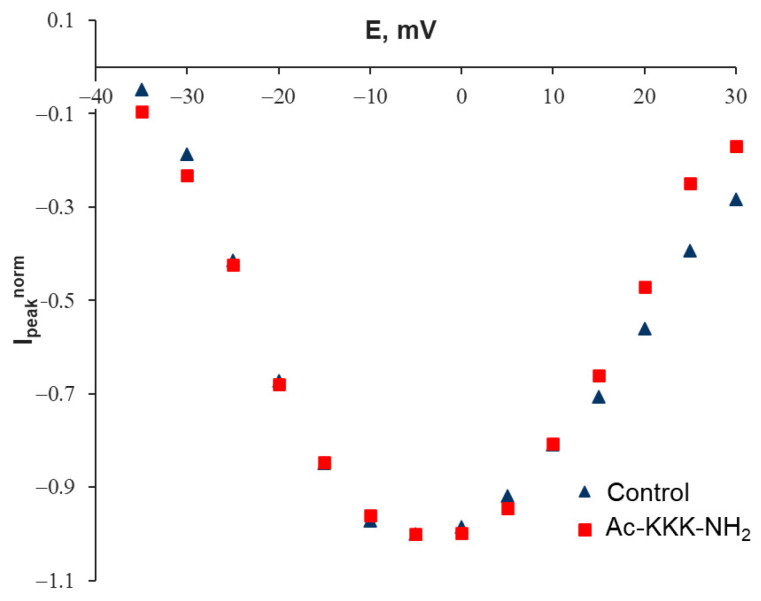
Normalized peak current–voltage characteristics of the Na_V_1.8 channels’ G_Na_(E)^norm^ in the control experiment and after extracellular application of Ac-KKK-NH_2_ at 100 nM.

The next step was the construction of the peak current–voltage characteristics of the Na_V_1.8 channels. The obtained current–voltage characteristics indicated a change in the steepness of the left branch of the function after exposure to the agent under study (Figure 3). This effect, observed at negative membrane potentials, made it possible to establish a quantitative relationship between the voltage sensitivity of the Na_V_1.8 channel activation gating system and the efficiency of binding of the attacking tripeptide to the Na_V_1.8 channel molecule.

**Figure 4:**
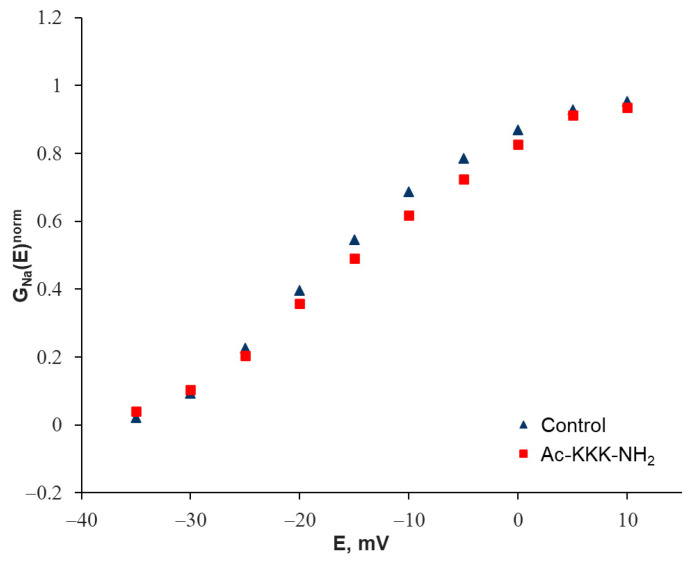
Normalized voltage sensitivity of the Na_V_1.8 channel chord conductance obtained in the control experiment and after extracellular application of Ac-KKK-NH_2_ at 100 nM.

More explicitly, the registered change in the steepness of the Na_V_1.8 channel peak current–voltage characteristics can be represented on the graph of the voltage dependence of the normalized chord conductance G_Na_(E)^norm^. This function was built as the ratio of the maximum amplitude of the Na_V_1.8 current to the difference between the membrane potential and the reversal potential of the Na_V_1.8 current. The G_Na_(E) function has an initial S-shaped section, the steepness of which reflects the characteristic features of the voltage sensitivity of the Na_V_1.8 channel activation process. When the normalized G^norm^_Na_(E) function was constructed, the change in the steepness of this particular section after the Ac-KKK-NH_2_ application was clearly manifested (Figure 4).

**Figure 5:**
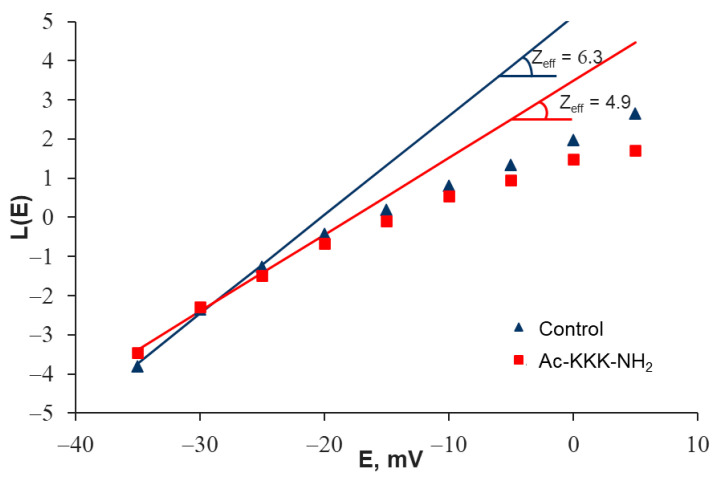
Evaluation of the Z_eff_ transferred by the Na_V_1.8 channel activation gating system using the Almers logarithmic limiting voltage sensitivity L(E) function in the control experiment and after extracellular application of Ac-KKK-NH_2_ at 100 nM.

**Figure 6:**
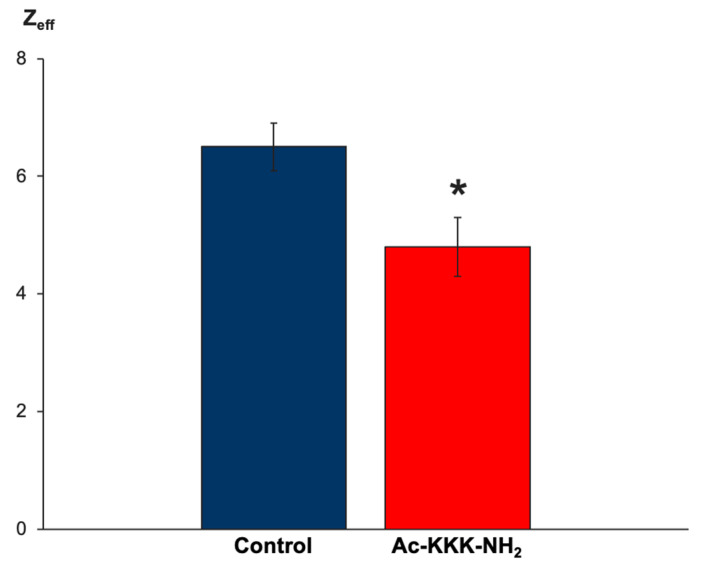
The value of the Z_eff_ transferred by the Na_V_1.8 channel activation gating system decreased from 6.5 ± 0.4 (n = 25) in the control experiments to 4.8 ± 0.5 (n = 25) after extracellular application of Ac-KKK-NH_2_ at 100 nM. Data are presented as mean ± SEM. Statistically significant difference between the control and experimental Z_eff_ values is designated with the asterisk (*p* < 0.05).

As mentioned earlier, the process of ligand–receptor binding of Ac-KKK-NH_2_ to the Na_V_1.8 channel is manifested in a change in the Z_eff_ value. To determine it, the modified Almers method was implemented by constructing the Almers logarithmic voltage sensitivity function L(E) (Figure 5). A series of experiments demonstrated a statistically significant (4.8 ± 0.5 (n = 25) vs. 6.5 ± 0.4 (n = 25)) decrease in the Z_eff_ value of the Na_V_1.8 channel activation gating system after extracellular application of Ac-KKK-NH_2_ at 100 nM (Figure 6). This result makes it possible to predict the analgesic effect of the tripeptide at the organismal level, which is verified further using the formalin test.

### 3.2. Organotypic Tissue Culture

**Figure 7:**
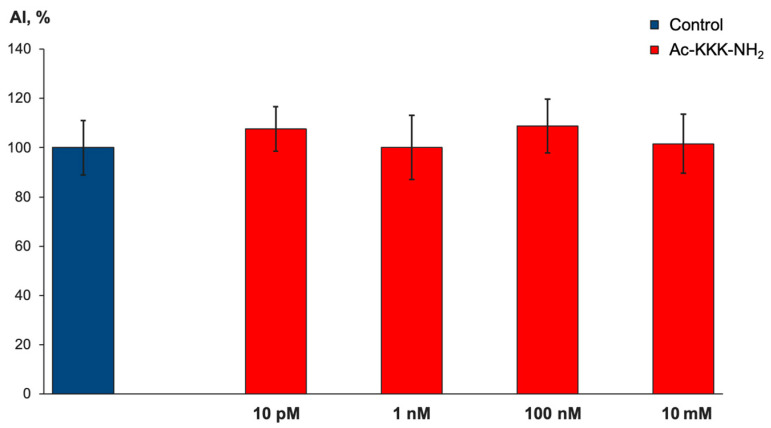
Effect of Ac-KKK-NH_2_ on DRG neurite growth. The ordinate axis—area index (AI, %). Data are presented as mean ± SEM (n = 29 for all groups, not significant, *p* > 0.5).

The organotypic tissue culture method was used to investigate the Ac-KKK-NH_2_ effect on DRG neurite growth. DRG explants were cultured with Ac-KKK-NH_2_ at 10 pM, 1 nM, 100 nM, and 10 mM for three days. At all studied concentrations, the area index (AI) of the experimental DRG explants did not significantly differ from the control value (Figure 7), which indicates that the tripeptide application neither promotes nor suppresses the growth of the nerve tissue under study.

### 3.3. Formalin Test

**Figure 8:**
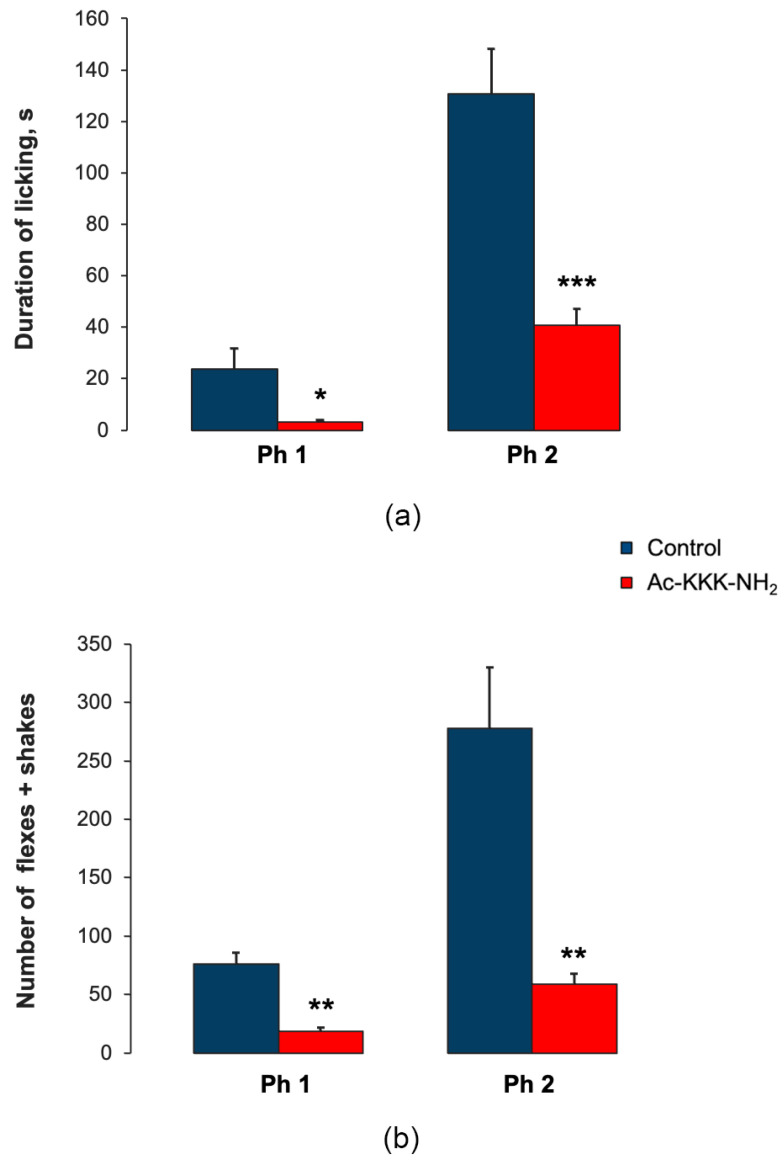
Effect of Ac-KKK-NH_2_ on licking duration and the number of flexes + shakes in the formalin test. (**a**) Licking duration in the acute (Ph1) and tonic (Ph2) phases; (**b**) the number of flexes + shakes in the acute (Ph1) and tonic (Ph2) phases. Data are presented as mean ± SEM. Statistically significant differences between the control and experimental values are designated with asterisks (* *p* < 0.05, ** *p* < 0.01, *** *p* < 0.001).

In the formalin test, the analgesic effect of Ac-KKK-NH_2_ at 1.0 mg/kg was demonstrated both at the spinal and supraspinal levels (Figure 8). At the supraspinal level, the administration of the tripeptide resulted in a statistically significant decrease in licking duration as compared with the control in both the acute (3.0 ± 1.0 vs. 23.8 ± 8.1, t = 2.555, *p* = 0.025) and tonic phases (40.5 ± 17.8 vs. 130.8 ± 6.4, t = 4.765, *p* = 0.00045) (Figure 8a). At the spinal level, a statistically significant decrease in the number of flexes + shakes both during the acute (18.6 ± 3.6 vs. 75.8 ± 10.2, t = 5.269, *p* = 0.0019) and tonic phases (59 ± 8.9 vs. 278 ± 52.2, t = 4.134, *p* = 0.0014) compared with the control was observed (Figure 8b).

### 3.4. Conformational Analysis

The Ac-KKK-NH_2_ tripeptide has a total charge of +3, as each of the lysine side chain amino groups bears a single positive charge. The N-terminal amino group is acylated and the C-terminal carboxylic group is amidated to rule out the possibility of electrostatic interaction between the charged terminal functional groups and the lysine amino groups and, at the same time, to protect the peptide from the destructive peptidases upon its administration in vivo. Hence, there are no other charged functional groups present in the Ac-KKK-NH_2_ than the lysine amino groups.

**Figure 9:**
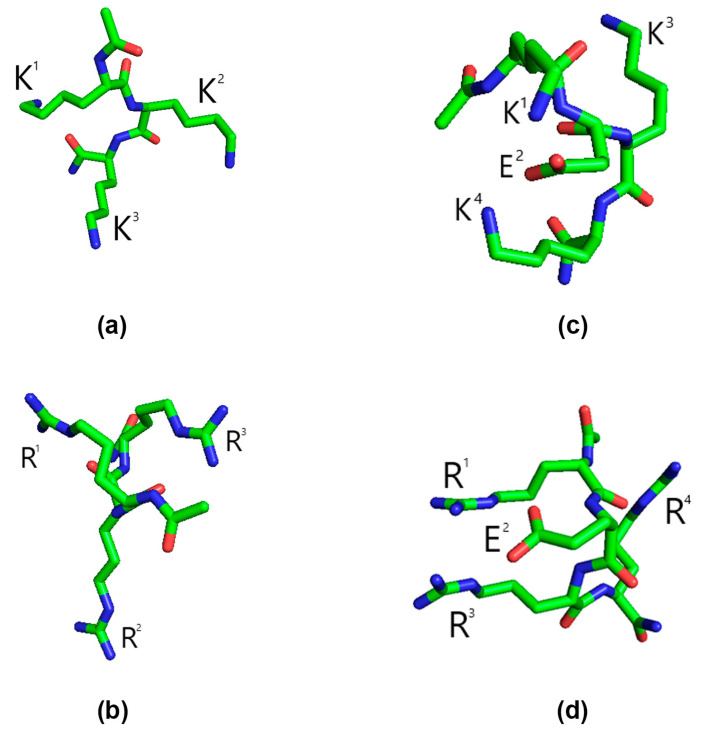
Lowest energy conformations of the peptides obtained by conformational analysis. (**a**) Ac-KKK-NH_2_; (**b**) Ac-RRR-NH_2_; (**c**) Ac-KEKK-NH_2_; (**d**) Ac-RERR-NH_2_. The dielectric constant ε = 10. Carbon, green; oxygen, red; nitrogen, blue. Hydrogen atoms are not shown. Data for Ac-RRR-NH_2_, Ac-KEKK-NH_2_, and Ac-RERR-NH_2_ are taken from [9,10,18].

The obtained results indicate that the lowest energy conformations and the average values of distances between the amino groups in all selected subensembles, as well as in the entire ensemble of ~100,000 conformations, are very much the same when calculated at ε = 10 and ε = 80; thus, only the data related to ε = 10 are presented and discussed further. The lowest energy conformation of Ac-KKK-NH_2_ is shown in Figure 9, which also includes, for comparison, the lowest energy conformations of Ac-RRR-NH_2_, Ac-KEKK-NH_2_, and Ac-RERR-NH_2_. These short peptides have also been shown to statistically significantly decrease the Z_eff_ of the Na_V_1.8 channel activation gating system at 100 nM [9,10,18], and one of them, Ac-KEKK-NH_2_, has been demonstrated in the formalin test to have an analgesic effect in vivo [18]. The values of distances between the lysine amino groups of Ac-KKK-NH_2_ averaged over the entire ensemble and over a number of smaller subensembles together with the corresponding values of distances between the charged functional groups in the molecules of Ac-RRR-NH_2_, Ac-KEKK-NH_2_, and Ac-RERR-NH_2_ are shown in Table 1.

We have earlier suggested that an ensemble required to realistically estimate the distances between the charged side chain functional groups in the low-energy conformational space of short lysine- and arginine-containing peptides should include at least 1% of the total count of conformations; that is, not less than a thousand [9,10,18]. Correspondingly, the average values of distances between the Ac-KKK-NH_2_ amino groups are obtained at the 4 kcal/mol energy cutoff (Table 1). The same energy cutoff has been obtained earlier for Ac-RRR-NH_2_ [10], whereas the cutoff value for Ac-KEKK-NH_2_ and Ac-RERR-NH_2_ has been determined to be 6 kcal/mol [9,18].

In the Ac-KKK-NH_2_ molecule, the K^1^–K^2^ and K^2^–K^3^ distances are fairly constant in all subensembles, while the K^1^–K^3^ distance decreases by ~1 Å with the decrement in the energy cutoff to 4 kcal/mol. This result is rather inevident, because the overall structure of the Ac-KKK-NH_2_ molecule is expected to be determined by electrostatic repulsion between the positively charged amino groups, which should drive them away from each other rather than bring them closer, as can be seen from the data presented. Comparing the data obtained herein with our earlier data for Ac-RRR-NH_2_, Ac-KEKK-NH_2_, and Ac-RERR-NH_2_ [9,10,18], a similar trend can be traced for the other short peptides: the distances between the first and the last charged functional groups decrease with the decrement in the energy cutoff value, while the distances between the middle functional group and any of the other two groups remain rather constant irrespective of the energy cutoff value. It can also be seen that the amino groups in both Ac-KKK-NH_2_ and Ac-KEKK-NH_2_ form almost equilateral triangles with the side lengths of ~11, 11, and 12 Å. It is worth noting that the charged functional groups in Ac-RRR-NH_2_ and Ac-RERR-NH_2_ form a somewhat distorted triangle due to possible stacking between the first and the last guanidinium groups.

The dielectric constant ε = 10. The distances between the amino groups in the lysine-containing peptides are calculated as the distances between the nitrogen atoms, and the distances between the guanidinium groups in the arginine-containing peptides are calculated as the distances between the central carbon atoms of the corresponding groups. Data for Ac-RRR-NH2, Ac-KEKK-NH2, and Ac-RERR-NH2 are taken from [9,10,18].

### 3.5. Peptide Docking with the Na_V_1.8 Channel Molecule

**Figure 10:**
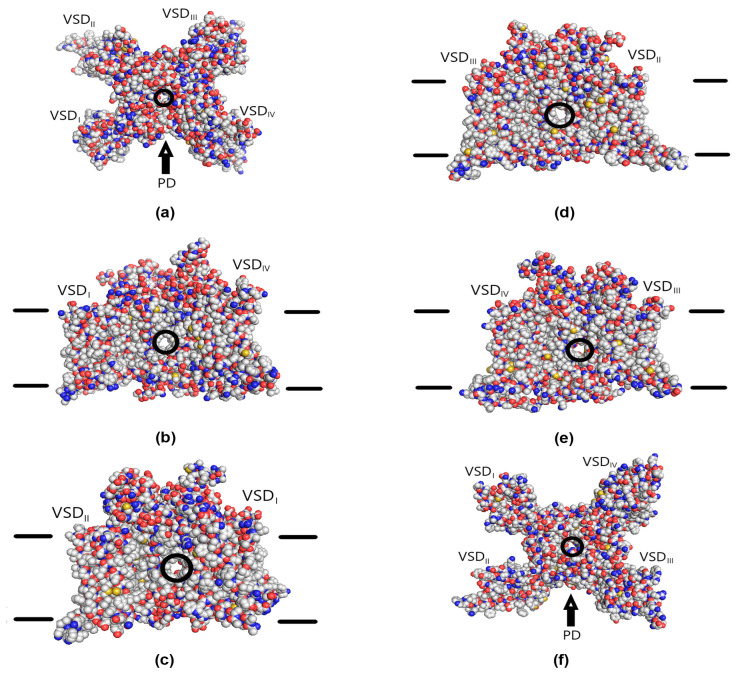
Structure of the Na_V_1.8 channel molecule. The extracellular (**a**), lateral (**b**–**e**), and intracellular (**f**) views. Voltage-sensing domains (VSD_I_-VSD_IV_) and pore domain (PD) are indicated. Carbon atoms, gray spheres; oxygen atoms, red spheres; nitrogen atoms, blue spheres; sulfur atoms, yellow spheres; hydrogen atoms not shown. The pore and the lateral fenestrations are highlighted with the black circles. The approximate positions of the membrane surfaces indicated by the black lines are taken from [27].

The extracellular, lateral, and intracellular views of the Na_V_1.8 channel molecule are presented in Figure 10. Somewhat simplified, the Na_V_1.8 channel contains the ion-permeating pore domain (PD) and four voltage-sensing domains (VSD_I_-VSD_IV_) that concertedly regulate the effective charge of the activation gating system. Approximately in the middle of the pore, there is a hydrophobic cavity that can also be accessed through the fenestrations located on the lateral sides of the pore domain between the voltage-sensing domains.

The average values of distances between the lysine side chain amino groups in the low-energy conformational space of the Ac-KKK-NH_2_ molecule calculated herein at ε = 10 with the energy cutoff value of 4 kcal/mol are as follows: μ_12_ = 10.8 Å, μ_13_ = 11.8 Å, μ_23_ = 10.6 Å (see Table 1). In accordance with the RMSD ˂ 1.5 criterion, 460 conformations of the tripeptide were selected from the 7 kcal/mol cutoff subensemble (15,132 conformations in total) for the further docking with the Na_V_1.8 channel. As each of the conformations was docked 4 times, 1840 single docking procedures have been performed. Almost 40% of them, 725, did not result in Ac-KKK-NH_2_ docking with the Na_V_1.8 channel molecule. Most probably, it is due to the difficulty locating a binding site that would effectively accommodate three positive amino group point charges at the same time.

**Figure 11:**
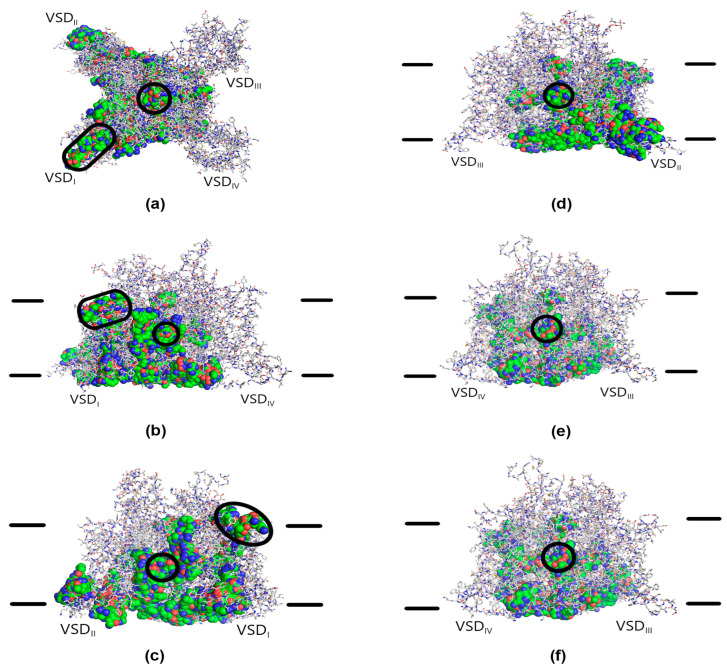
Superimposition of the docked Ac-KKK-NH_2_ conformations on the Na_V_1.8 channel molecule. The extracellular (**a**), lateral (**b**–**e**), and intracellular (**f**) views. Voltage-sensing domains (VSD_I_–VSD_IV_) are indicated. The Na_V_1.8 channel molecule is presented with the sticks. Carbon atoms, gray sticks; oxygen atoms, red sticks; nitrogen atoms, blue sticks; sulfur atoms, yellow sticks. The docked conformations are presented with the spheres. Carbon atoms, green spheres; oxygen atoms, red spheres; nitrogen atoms, blue spheres. Hydrogen atoms not shown. The pore and the lateral fenestrations are highlighted with the black circles. The extracellular VSD_I_ binding site is highlighted with the black ovals. The approximate positions of the membrane surfaces indicated by the black lines are taken from [27].

The results of 1115 successful Ac-KKK-NH_2_ docking procedures are presented in Figure 11 as the superimposition of the obtained peptide–channel complexes. On the extracellular side of the Na_V_1.8 channel molecule, two sparsely populated binding sites were located: 15 conformations docked to VSD_I_ and another 15 conformations docked to PD at the pore entrance. On the lateral sides, the tripeptide docked to the interfaces between PD and VSD_I_ on both sides of the latter and to the lateral surface of PD between VSD_I_ and VSD_II_ (151 conformations in total). The attacking peptide molecule also docked to the intracellular surface of the Na_V_1.8 channel in the area of PD, VSD_I,_ and VSD_II_ (197 conformations in total). A substantial number of conformations (312) docked into the hydrophobic inner cavity and the hydrophobic fenestrations leading to it from the lateral sides of the Na_V_1.8 channel molecule. The most populated binding site (367 conformations) was located within the intracellular part of VSD_I_, while another 58 conformations docked to the intracellular part of VSD_II_. It is worth mentioning that no binding sites were detected on either VSD_III_ or VSD_IV_.

The existence of the neuron membrane was not taken into account during the docking procedure. When the approximate membrane position in physiologically adequate conditions is considered (Figure 11b–e), it turns out that most of the detected binding sites are technically inaccessible for the Ac-KKK-NH_2_ molecule without penetrating the membrane lipid bilayer, with the exception of the two sparsely populated ones located within the extracellular part of VSD_I_ and at the extracellular entrance to the channel pore. However, binding of the tripeptide to the pore entrance would block the sodium current, which makes the extracellular part of VSD_I_ the only binding site accessible for the attacking molecule.

The observed location of inaccessible binding sites is rather expected given the necessity to effectively accommodate three positive amino group point charges. The tripeptide mostly docked either into the hydrophobic cavity and its fenestrations or to the channel surface from the intracellular and lateral sides. Of some interest are the two binding sites located within the intracellular parts of VSD_I_ and VSD_II_, because some of the docked conformations interact with the lower part of the voltage sensor in these domains. Still, the transmembrane Ac-KKK-NH_2_ penetration would be required to reach these binding sites in physiologically adequate conditions.

**Figure 12:**
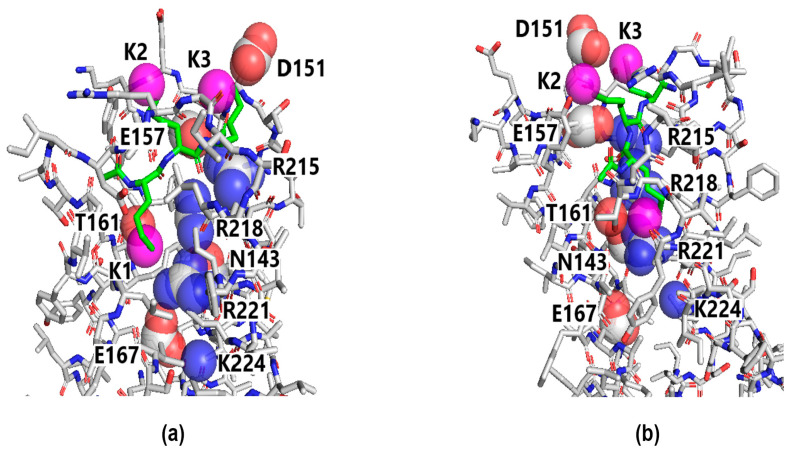
Detailed view of the Ac-KKK-NH_2_ binding site located on the extracellular part of VSD_I_. Two lateral projections, normal to each other (**a**,**b**). The VSD_I_ domain is presented with the sticks. Carbon atoms, gray sticks; oxygen atoms, red sticks; nitrogen atoms, blue sticks. The bound Ac-KKK-NH_2_ molecule is also shown with the sticks. Carbon atoms, green sticks; oxygen atoms, red sticks; nitrogen atoms, blue sticks. Hydrogen atoms not shown. The guanidinium groups of Arg215 (R215), Arg218 (R218), Arg221 (R221), the amino group of Lys224 (K224), the carboxylate groups of Asp151 (D151), Glu157 (E157), Glu167 (E167), the hydroxyl group of Thr161 (T161), and the carboxamide group of Asn143 (N143) are highlighted with partially transparent spheres of the corresponding colors. The amino groups of Ac-KKK-NH_2_ are shown with the magenta spheres.

A more detailed view of the Ac-KKK-NH_2_ binding site located on the extracellular part of VSD_I_ is presented in Figure 12. The voltage sensor contains four positively charged amino acid residues Arg215, Arg218, Arg221, and Lys224 on the transmembrane segment S4_I_ that shifts upwards to the extracellular side of the neuron membrane upon the channel activation. In the considered form of the Na_V_1.8 channel molecule with the voltage sensor in the “up” (or depolarized) state, Arg215 forms ionic bonds with Asp151 and Glu157, Arg218 is hydrogen-bonded to Thr161, Arg221 is hydrogen-bonded to Asn143, and Lys224 interacts with Glu167 [23]. The K2 and K3 amino groups of the bound Ac-KKK-NH_2_ molecule compete with Arg215 for Asp151 and Glu157, while the K1 amino group competes with Arg218 to form a hydrogen bond with Thr161. Therefore, the peptide binding is demonstrated to strongly influence the intramolecular bonding patterns within VSD_I,_ which attach the voltage sensor to the rest of the domain. As a result, the coordinated movement of the voltage sensor up and down is affected, thus modulating the value of the effective charge Z_eff_ transferred by the Na_V_1.8 channel activation gating system upon the channel opening.

## 4. Discussion

The main result of this work is the discovery of a possible molecular mechanism of the short lysine-containing tripeptide Ac-KKK-NH_2_ ligand–receptor binding to the Na_V_1.8 channel molecule. The research methodology applied herein combines experimental physiological and theoretical calculational techniques that made it possible to locate the Ac-KKK-NH_2_ binding site on the Na_V_1.8 channel activation gating system, namely, on the voltage-sensing domain VSD_I_. According to the results obtained, two of the amino groups of the attacking peptide form intermolecular ionic bonds with Asp151 and Glu157 close to the Na_V_1.8 channel extracellular surface, while the third amino group is buried deeper within VSD_I_ and forms a hydrogen bond with Thr161. The indicated amino acid residues also interact with Arg215 and Arg218, two of the four positively charged residues that belong to the voltage sensor of VSD_I_ and directly regulate the contribution of this domain to the total Na_V_1.8 channel gating charge. The Ac-KKK-NH_2_ binding requires effective accommodation of the three positively charged amino groups, which substantially affects the intramolecular bonding patterns that natively coordinate the positively charged amino acid residues of the VSD_I_ voltage sensor and thus provide its correct functioning. In addition to that, a noticeable spatial displacement of the voltage sensor is expected due to the electrostatic repulsion from the amino group point charges of the bound tripeptide. At the molecular level, this is what should account for the Ac-KKK-NH_2_-induced decrease in the Na_V_1.8 channel activation gating system effective charge, Z_eff_, detected in our patch-clamp experiments.

According to the Gouy–Chapman–Stern theory, the sodium gating mechanism is sensitive to the local transmembrane potential, which is different from the bulk-to-bulk membrane potential due to the surface charges on the outer side of the neuron membrane [10]. The peak values of the current–voltage characteristics both before and after the Ac-KKK-NH_2_ application are observed at E ≈ 0 (Figure 3), which indicates the specific and direct peptide binding to the Nav1.8 channel and not to any other membrane proteins coupled to this channel, because the current–voltage characteristics would otherwise shift along the E axis [7]. A decrease in the Z_eff_ value and the consequent decrease in the Na_V_1.8 channel functional activity at the cellular level unambiguously results in an antinociceptive reaction at the organismal level, which has been herein demonstrated in the formalin test in vivo.

The fact that Ac-KKK-NH_2_ applied in a wide range of concentrations does not affect the DRG neurite growth provides another piece of evidence for its direct specific interaction with the Na_V_1.8 channel, indicating that the tripeptide does not bind to the neighboring membrane proteins that activate intracellular signaling cascades. Otherwise, the neurite growth is affected, as has been demonstrated by the very sensitive organotypic tissue culture method for the other substances that modulate the Nav1.8 channel voltage sensitivity by the receptor- or transducer-coupled mechanisms [7,8,14].

Theoretical calculational methods can provide an insight into the structure of the Ac-KKK-NH_2_—Na_V_1.8 channel ligand–receptor complex at the atomic level, which allowed us to gain a deeper knowledge of the voltage sensor functioning mechanism and determine the amino acid residues of the Na_V_1.8 channel molecule that coordinate the positively charged functional groups of the bound tripeptide. Therefore, the point mutations involving Asp151, Glu157, and Thr161 are expected to affect both the characteristics of the VSD_I_ voltage sensor and the Ac-KKK-NH_2_ binding, which could result in severe damage to the nociceptive system, possibly leading to hyperalgesia, allodynia, and other neuropathological disfunctions. The importance of conducting such studies for understanding the fundamental mechanisms of nerve tissue excitability is difficult to overestimate. As has been demonstrated in the present work and earlier [9,10,18], the lysine- and arginine-containing short peptides that decrease the Na_V_1.8 channel activation gating system effective charge at 100 nM should contain three positively charged functional groups forming an almost equilateral triangle with the side length of 10–12 Å; otherwise, the attacking peptides were not active.

The main goal of the conformational analysis is to correlate the distances between the charged functional groups in a specific conformation with the energy of this conformation. Averaging the distances over a number of subensembles defined by different energy cutoff values, as well as over the entire conformational ensemble of 100,000 conformations, has made it possible to determine the distances in the low-energy conformational space of the studied peptides. These values were used further to select the low-energy conformations for the docking. In other words, the conformations that did not match the obtained distance criteria had been taken out of consideration, because they were supposed to bind to the Na_V_1.8 channel less effectively, if at all. It is difficult to comment on the Na_V_1.8 channel conformational changes under physiological conditions, as just a single Na_V_1.8 channel structural model published very recently is available [23].

All low-energy Ac-KKK-NH_2_ conformations obtained in the conformational analysis, the positions of the amino groups that do not substantially deviate from the abovementioned triangle, have been blindly docked herein with the Na_V_1.8 channel molecule. This approach has made it possible to determine the only physiologically relevant Ac-KKK-NH_2_ binding site located on the VSD_I_ domain, which correlates with the decrease in the Z_eff_ value observed in our patch-clamp experiments. It is important to note that none of the selected conformations docked with any other VSD from the extracellular side, which suggests a VSD_I_-selective Ac-KKK-NH_2_ binding. In addition, VSD_I_ in Na_V_1.8 has been demonstrated to be conformationally flexible, which appears to be the only major variation between the structures of Na_V_1.8 and other Na_v_ channels [23]. It is thus tempting to predict a subtype- and domain-specific targeting of the short lysine- and arginine-containing peptides on VSD_I_ in the Na_V_1.8 channels, which makes them promising candidates for the role of novel, safe (due to their endogenous nature), and very effective analgesic medicinal substances that are expected to be of great practical significance. In current clinical practice, there are no medicines that specifically modulate the Na_V_1.8 channels. Since morphine has been shown to decrease the Na_V_1.8 channel voltage sensitivity [7,28], it can serve as a reference agent to contextualize the clinical potential of Ac-KKK-NH_2_. Moreover, tripeptide is absolutely safe due to its direct interaction with the Na_V_1.8 channel activation gating system and its endogenous nature, as opposed to the exogenous morphine binding to the opioid receptors that activate G-protein-mediated signaling. Our in vivo experiments provide solid evidence for the strong Ac-KKK-NH_2_ analgesic effect at both the spinal and the supraspinal levels.

It is worth pointing out that the same indicated peptide concentration of 100 nM has been used in the patch-clamp experiments both herein and in our prior publications [9,10,18], which allowed us to bridge the gap between research at the molecular level and in vivo experiments. It has turned out that the concentration of 100 nM in the patch-clamp experiments corresponds to the dose of ~1.0 mg/kg in the formalin test that evokes a strong analgesic effect at both the spinal and supraspinal levels. This observation has been confirmed herein and when studying another lysine-containing peptide, Ac-KEKK-NH_2_ [18]. Changing the chosen concentrations is not expected to substantially improve the analgesic properties of the studied peptides, while it might result in adverse side effects when elevating the peptide concentration or in the loss of the observed analgesic effect when lowering the peptide concentration.

## 5. Conclusions

There are absolutely no safe big analgesics in the arsenal of practical medicine, the use of which could be compared with opiates in their effectiveness. In our opinion, the short peptide Ac-KKK-NH_2_, a substance of endogenous nature, could fill this gap, which has been demonstrated in the present work using a complex of modern experimental and theoretical methods. This tripeptide induces a strong analgesic effect at both the spinal and supraspinal levels, as confirmed by in vivo experiments in the formalin test. Moreover, the Ac-KKK-NH_2_ action is direct and specific to the Na_V_1.8 channel, the evidence being provided by the absence of the shift in the peak current–voltage characteristics along the voltage axis after its application in the patch-clamp experiments and by the absence of its effect in a wide range of concentrations on the DRG neurite growth in the embryonic nervous tissue, which is extremely sensitive to any substances of exogenous and endogenous nature. Our results indicate that this specificity is based on the subtle mechanism of ligand–receptor binding of the tripeptide to the voltage-sensing domain VSD_I_ of the Na_V_1.8 channel activation gating system. These channels are known markers of nociceptive neurons, whose high-frequency impulse firing transmits information about painful (damaging) stimuli to the central nervous system. The conformational analysis has made it possible to determine that the positively charged amino groups form an almost equilateral triangle with the side lengths of ~11–12 Å in the low-energy conformational space of the Ac-KKK-NH_2_ molecule. Finally, blind docking of the tripeptide to the Na_V_1.8 channel has revealed that the amino groups of the attacking peptide interact with the Asp151, Glu157, and Thr161 amino acid residues of VSD_I_. We suggest that the tripeptide binding results in a decrease in the Na_V_1.8 channel voltage sensitivity observed in the patch-clamp experiments and, consequently, turns off the high-frequency (pronociceptive) component of the responses of polymodal tissue receptors. It can also be assumed that point mutations of Asp151, Glu157, and Thr161 in the native Na_V_1.8 channel structure might drastically affect the fundamental mechanism of the somatosensory antinociceptive reaction at the organismal level.

## Figures and Tables

**Table 1:** Average distances between the charged side chain functional groups in the molecules of short lysine- and arginine-containing peptides.

Cutoff, kcal/mol	Ac-KKK-NH_2_		Ac-RRR-NH_2_		Ac-KEKK-NH_2_		Ac-RERR-NH_2_	
	N_conf_	Distances, Å	N_conf_	Distances, Å	N_conf_	Distances, Å	N_conf_	Distances, Å
None	103,478	K^1^–K^2^ 10.9 ± 2.4	102,764	R^1^–R^2^ 10.7 ± 2.6	102,834	K^1^–K^3^ 11.8 ± 2.9	101,546	R^1^–R^3^ 9.8 ± 3.4
		K^1^–K^3^ 12.7 ± 3.1		R^1^–R^3^ 11.1 ± 3.7		K^1^–K^4^ 12.8 ± 3.7		R^1^–R^4^ 9.2 ± 3.7
		K^2^–K^3^ 10.7 ± 2.3		R^2^–R^3^ 11.0 ± 2.7		K^3^–K^4^ 10.7 ± 2.1		R^3^–R^4^ 9.4 ± 2.7
7	15,132	K^1^–K^2^ 11.0 ± 2.2	15,428	R^1^–R^2^ 10.6 ± 2.6	3778	K^1^–K^3^ 12.0 ± 2.8	2354	R^1^–R^3^ 9.7 ± 3.1
		K^1^–K^3^ 12.3 ± 3.0		R^1^–R^3^ 9.6 ± 3.4		K^1^–K^4^ 11.3 ± 3.4		R^1^–R^4^ 8.0 ± 2.8
		K^2^–K^3^ 10.8 ± 2.2		R^2^–R^3^ 10.9 ± 2.6		K^3^–K^4^ 11.0 ± 1.7		R^3^–R^4^ 9.1 ± 2.4
6	9732	K^1^–K^2^ 11.0 ± 2.2	9798	R^1^–R^2^ 10.4 ± 2.6	1551	K^1^–K^3^ 11.9 ± 2.7	1023	R^1^–R^3^ 9.7 ± 3.1
		K^1^–K^3^ 12.1 ± 3.0		R^1^–R^3^ 9.3 ± 3.3		K^1^–K^4^ 11.1 ± 3.3		R^1^–R^4^ 8.2 ± 2.7
		K^2^–K^3^ 10.8 ± 2.2		R^2^–R^3^ 10.9 ± 2.5		K^3^–K^4^ 10.9 ± 1.7		R^3^–R^4^ 9.2 ± 2.3
5	5263	K^1^–K^2^ 10.9 ± 2.2	5197	R^1^–R^2^ 10.3 ± 2.6	579	K^1^–K^3^ 11.7 ± 2.7	408	R^1^–R^3^ 9.3 ± 3.2
		K^1^–K^3^ 12.0 ± 3.0		R^1^–R^3^ 9.0 ± 3.0		K^1^–K^4^ 11.0 ± 3.3		R^1^–R^4^ 8.2 ± 2.4
		K^2^–K^3^ 10.7 ± 2.1		R^2^–R^3^ 11.0 ± 2.4		K^3^–K^4^ 10.9 ± 1.7		R^3^–R^4^ 9.3 ± 2.3
4.5	3433	K^1^–K^2^ 10.9 ± 2.2	3498	R^1^–R^2^ 10.2 ± 2.6	351	K^1^–K^3^ 11.6 ± 2.7	258	R^1^–R^3^ 9.1 ± 3.3
		K^1^–K^3^ 12.0 ± 2.9		R^1^–R^3^ 8.8 ± 2.8		K^1^–K^4^ 10.8 ± 3.3		R^1^–R^4^ 8.1 ± 2.4
		K^2^–K^3^ 10.7 ± 2.0		R^2^–R^3^ 11.0 ± 2.4		K^3^–K^4^ 10.8 ± 1.8		R^3^–R^4^ 9.4 ± 2.2
4	2046	K^1^–K^2^ 10.8 ± 2.1	2247	R^1^–R^2^ 10.2 ± 2.6	186	K^1^–K^3^ 10.8 ± 2.7	157	R^1^–R^3^ 8.6 ± 3.2
		K^1^–K^3^ 11.8 ± 2.9		R^1^–R^3^ 8.7 ± 2.7		K^1^–K^4^ 10.9 ± 3.3		R^1^–R^4^ 8.2 ± 2.4
		K^2^–K^3^ 10.6 ± 2.0		R^2^–R^3^ 11.1 ± 2.4		K^3^–K^4^ 10.8 ± 1.8		R^3^–R^4^ 9.5 ± 2.2
3	500	K^1^–K^2^ 10.6 ± 2.0	790	R^1^–R^2^ 10.1 ± 2.6	55	K^1^–K^3^ 10.4 ± 2.7	55	R^1^–R^3^ 7.6 ± 2.9
		K^1^–K^3^ 11.3 ± 2.6		R^1^–R^3^ 8.5 ± 2.8		K^1^–K^4^ 10.6 ± 3.2		R^1^–R^4^ 8.7 ± 2.1
		K^2^–K^3^ 10.6 ± 1.6		R^2^–R^3^ 11.4 ± 2.1		K^3^–K^4^ 11.0 ± 1.5		R^3^–R^4^ 9.9 ± 2.0

## Data Availability

Data are contained within the article and Supplementary Materials.

## Supplementary Materials

The following supporting information can be downloaded at: https://www.mdpi.com/article/10.3390/life14101337/s1, Figure S1: Families of slow sodium Na_V_1.8 currents in the control experiment (above) and after extracellular application of Ac-KKK-NH_2_ at 100 nM (below).
