# Supplementary material for: Short Lysine-Containing Tripeptide as Analgesic Substance: The Possible Mechanism of Ligand–Receptor Binding to the Slow Sodium Channel
Source: Life (Basel). 2024 Oct 21;14(10):1337. doi: 10.3390/life14101337 (PMC11509431; doi:10.3390/life14101337)
Supplement: Supplementary file 1 [file life-14-01337-s001.zip › life-3215904-supplementary.pdf]

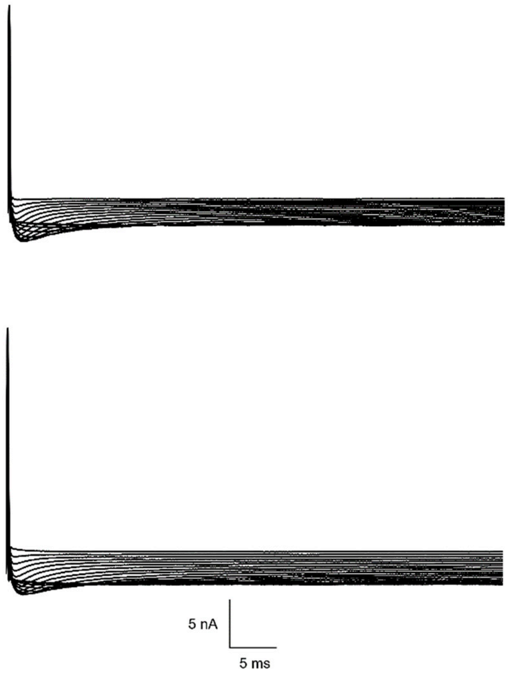

Figure S1. Families of slow sodium $Na_V1.8$ currents in the control experiment (above) and after extracellular application of Ac-KKK-NH_2 at 100 nM (below).
